# Methyl 5-bromo-6-methyl­picolinate

**DOI:** 10.1107/S1600536808042104

**Published:** 2008-12-17

**Authors:** Ya-Ming Wu, Chun-Ming Wu, Yong Wang

**Affiliations:** a Department of Applied Chemistry, Nanjing College of Chemical Technology, No. 625 Geguan Road, Dachang, Nanjing 210048, People’s Republic of China; b Synergetica-Changzhou, Ltd., Chunjiang Town, Xinbei District, Changzhou City 213033, Jiangsu Province, People’s Republic of China; c School of Pharmaceutical and Chemical Engineering, Taizhou University, Linhai 317000, People’s Republic of China

## Abstract

The title compound, C_8_H_8_BrNO_2_, does not show any significant inter­molecular π–π or C—H⋯π inter­actions in the crystal packing except for one weak Br⋯Br [3.715 (1) Å] inter­action.

## Related literature

The title compound is an important inter­mediate for the construction of novel supported PyOX ligands, see: Oila *et al.* (2005 ▶). For bond-length data, see: Allen *et al.* (1987 ▶).
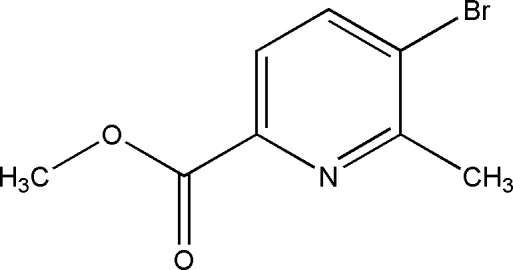


## Experimental

### 

#### Crystal data

C_8_H_8_BrNO_2_
*M* *_r_* = 230.06
Monoclinic, 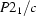

*a* = 18.518 (4) Å
*b* = 4.1040 (8) Å
*c* = 12.442 (3) Å
β = 109.52 (3)°
*V* = 891.2 (4) Å^3^
*Z* = 4
Mo *K*α radiation
μ = 4.57 mm^−1^
*T* = 293 (2) K
0.20 × 0.10 × 0.10 mm

#### Data collection

Enraf–Nonius CAD-4 diffractometer
Absorption correction: ψ scan (North *et al.*, 1968 ▶) *T*_min_ = 0.462, *T*_max_ = 0.658
1602 measured reflections
1602 independent reflections
975 reflections with *I* > 2σ(*I*)
3 standard reflections every 200 reflections intensity decay: 1%

#### Refinement

*R*[*F* ^2^ > 2σ(*F* ^2^)] = 0.061
*wR*(*F* ^2^) = 0.066
*S* = 1.75
1602 reflections
110 parameters
H-atom parameters constrained
Δρ_max_ = 0.53 e Å^−3^
Δρ_min_ = −0.61 e Å^−3^

### 

Data collection: *CAD-4 Software* (Enraf–Nonius, 1985 ▶); cell refinement: *CAD-4 Software*; data reduction: *XCAD4* (Harms & Wocadlo, 1995 ▶); program(s) used to solve structure: *SHELXS97* (Sheldrick, 2008 ▶); program(s) used to refine structure: *SHELXL97* (Sheldrick, 2008 ▶); molecular graphics: *SHELXTL* (Sheldrick, 2008 ▶); software used to prepare material for publication: *SHELXTL*.

## Supplementary Material

Crystal structure: contains datablocks I, global. DOI: 10.1107/S1600536808042104/lx2083sup1.cif

Structure factors: contains datablocks I. DOI: 10.1107/S1600536808042104/lx2083Isup2.hkl

Additional supplementary materials:  crystallographic information; 3D view; checkCIF report

## Figures and Tables

**Fig. 1.**
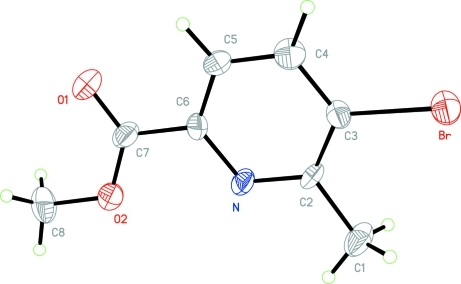
A drawing of the title molecular structure, with the atom-numbering scheme. Displacement ellipsoids are drawn at the 30% probability level.

**Fig. 2.**
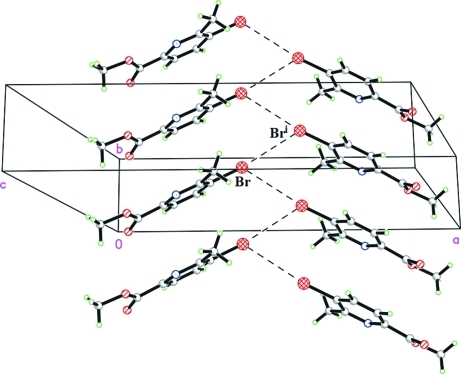
Br···Br interaction in the title compound. [Symmetry code: (i) -x+1, y+1/2, -z+1/2.]

## supplementary crystallographic information

### Comment

The title compound is one of important intermediates for construction of novel supported PyOX-ligands (Oila *et al.*,  2005). Here we report the crystal structure of the title compound, methyl 5-bromo-6-methylpicolinate (Fig. 1).

In the title compound, the bond lengths and angles are within normal ranges (Allen *et al.*,  1987). The crystal structure is stabilized by a weak Br···Br^i^ interaction at 3.715 (1) Å (Fig. 2; symmetry code as in Fig. 2).

### Experimental

The title compound, (I) was prepared by a method reported in literature (Oila *et al.*, 2005) with some modification. The crystals were obtained by dissolving I (0.2 g) in methanol (50 ml) and evaporating the solvent slowly at room temperature for about 3 d.

### Refinement

H atoms were positioned geometrically, with O—H = 0.82 and C—H = 0.93 Å for aromatic H, and constrained to ride on their parent atoms, with *U*_iso_(H) = *xU*_eq_(C/O), where *x* = 1.2 for aromatic H and *x* = 1.5 for other H.

### Crystal data

**Table:**

C_8_H_8_BrNO_2_	*F*(000) = 456
*M**_r_* = 230.06	*D*_x_ = 1.715 Mg m^−^^3^
Monoclinic, *P*2_1_/*c*	Mo *K*α radiation, λ = 0.71073 Å
Hall symbol: -P 2ybc	Cell parameters from 25 reflections
*a* = 18.518 (4) Å	θ = 10–14°
*b* = 4.1040 (8) Å	µ = 4.57 mm^−^^1^
*c* = 12.442 (3) Å	*T* = 293 K
β = 109.52 (3)°	Block, colorless
*V* = 891.2 (4) Å^3^	0.20 × 0.10 × 0.10 mm
*Z* = 4	

### Data collection

**Table:**

Enraf–Nonius CAD-4 diffractometer	975 reflections with *I* > 2σ(*I*)
Radiation source: fine-focus sealed tube	*R*_int_ = 0.0000
graphite	θ_max_ = 25.3°, θ_min_ = 1.2°
ω/2θ scans	*h* = −22→20
Absorption correction: ψ scan (North *et al.*, 1968)	*k* = 0→4
*T*_min_ = 0.462, *T*_max_ = 0.658	*l* = 0→14
1602 measured reflections	3 standard reflections every 200 reflections
1602 independent reflections	intensity decay: 1%

### Refinement

**Table:**

Refinement on *F*^2^	Primary atom site location: structure-invariant direct methods
Least-squares matrix: full	Secondary atom site location: difference Fourier map
*R*[*F*^2^ > 2σ(*F*^2^)] = 0.061	Hydrogen site location: difference Fourier map
*wR*(*F*^2^) = 0.066	H-atom parameters constrained
*S* = 1.75	*w* = 1/[σ^2^(*F*_o_^2^)]
1602 reflections	(Δ/σ)_max_ < 0.000
110 parameters	Δρ_max_ = 0.53 e Å^−^^3^
0 restraints	Δρ_min_ = −0.61 e Å^−^^3^

### Special details

**Table:**

Geometry. All esds (except the esd in the dihedral angle between two l.s. planes) are estimated using the full covariance matrix. The cell esds are taken into account individually in the estimation of esds in distances, angles and torsion angles; correlations between esds in cell parameters are only used when they are defined by crystal symmetry. An approximate (isotropic) treatment of cell esds is used for estimating esds involving l.s. planes.
Refinement. Refinement of F^2^ against ALL reflections. The weighted R-factor wR and goodness of fit S are based on F^2^, conventional R-factors R are based on F, with F set to zero for negative F^2^. The threshold expression of F^2^ > 2sigma(F^2^) is used only for calculating R-factors(gt) etc. and is not relevant to the choice of reflections for refinement. R-factors based on F^2^ are statistically about twice as large as those based on F, and R- factors based on ALL data will be even larger.

### Fractional atomic coordinates and isotropic or equivalent isotropic displacement parameters (Å^2^)

**Table:**

	*x*	*y*	*z*	*U*_iso_*/*U*_eq_	
Br	0.41815 (4)	0.6507 (2)	0.25731 (6)	0.0546 (3)	
O1	0.0882 (2)	−0.1451 (13)	0.2125 (3)	0.0691 (16)	
O2	0.1349 (2)	−0.0435 (11)	0.3986 (4)	0.0533 (15)	
N	0.2635 (3)	0.2009 (15)	0.3797 (4)	0.0450 (17)	
C1	0.3893 (3)	0.4149 (17)	0.4811 (4)	0.067 (2)	
H1A	0.3707	0.3787	0.5434	0.101*	
H1B	0.4059	0.6370	0.4822	0.101*	
H1C	0.4317	0.2717	0.4882	0.101*	
C2	0.3260 (3)	0.3477 (18)	0.3698 (5)	0.0344 (16)	
C3	0.3290 (3)	0.4444 (16)	0.2646 (5)	0.039 (2)	
C4	0.2680 (3)	0.3789 (19)	0.1651 (5)	0.059 (2)	
H4A	0.2702	0.4358	0.0939	0.071*	
C5	0.2053 (3)	0.2298 (18)	0.1755 (5)	0.050 (2)	
H5A	0.1632	0.1866	0.1109	0.060*	
C6	0.2038 (3)	0.1425 (19)	0.2814 (5)	0.0426 (18)	
C7	0.1372 (4)	−0.0335 (18)	0.2958 (6)	0.050 (2)	
C8	0.0676 (3)	−0.1896 (19)	0.4127 (5)	0.070 (2)	
H8A	0.0724	−0.1875	0.4920	0.104*	
H8B	0.0627	−0.4104	0.3857	0.104*	
H8C	0.0230	−0.0679	0.3698	0.104*	

### Atomic displacement parameters (Å^2^)

**Table:**

	*U*^11^	*U*^22^	*U*^33^	*U*^12^	*U*^13^	*U*^23^
Br	0.0482 (4)	0.0590 (5)	0.0589 (5)	−0.0020 (6)	0.0211 (3)	0.0028 (6)
O1	0.048 (3)	0.092 (4)	0.055 (4)	−0.011 (4)	0.001 (3)	−0.013 (4)
O2	0.043 (3)	0.070 (4)	0.046 (3)	−0.007 (3)	0.013 (2)	−0.002 (3)
N	0.029 (3)	0.065 (5)	0.033 (3)	−0.006 (4)	0.000 (3)	0.004 (4)
C1	0.056 (4)	0.087 (7)	0.043 (4)	−0.027 (5)	−0.004 (4)	0.012 (5)
C2	0.026 (4)	0.031 (4)	0.035 (4)	−0.003 (4)	−0.004 (3)	−0.004 (5)
C3	0.034 (4)	0.045 (6)	0.040 (4)	0.002 (4)	0.014 (4)	0.009 (4)
C4	0.049 (4)	0.088 (7)	0.033 (4)	0.004 (6)	0.005 (4)	0.031 (5)
C5	0.038 (4)	0.076 (7)	0.029 (4)	0.001 (4)	0.002 (3)	0.002 (4)
C6	0.025 (4)	0.060 (5)	0.042 (4)	0.004 (5)	0.011 (3)	−0.001 (5)
C7	0.037 (5)	0.060 (6)	0.043 (5)	0.004 (4)	0.000 (4)	−0.010 (5)
C8	0.051 (4)	0.079 (7)	0.089 (5)	−0.011 (5)	0.037 (4)	0.010 (6)

### Geometric parameters (Å, °)

**Table:**

Br—Br^i^	3.715 (1)	C2—C3	1.387 (6)
Br—C3	1.884 (5)	C3—C4	1.396 (6)
O1—C7	1.218 (6)	C4—C5	1.356 (7)
O2—C7	1.294 (6)	C4—H4A	0.9300
O2—C8	1.446 (6)	C5—C6	1.375 (7)
N—C2	1.347 (6)	C5—H5A	0.9300
N—C6	1.368 (6)	C6—C7	1.491 (8)
C1—C2	1.510 (6)	C8—H8A	0.9600
C1—H1A	0.9600	C8—H8B	0.9600
C1—H1B	0.9600	C8—H8C	0.9600
C1—H1C	0.9600		
			
C7—O2—C8	116.6 (5)	C3—C4—H4A	121.0
C2—N—C6	117.3 (5)	C4—C5—C6	120.0 (6)
C2—C1—H1A	109.5	C4—C5—H5A	120.0
C2—C1—H1B	109.5	C6—C5—H5A	120.0
H1A—C1—H1B	109.5	N—C6—C5	122.9 (6)
C2—C1—H1C	109.5	N—C6—C7	115.5 (6)
H1A—C1—H1C	109.5	C5—C6—C7	121.6 (6)
H1B—C1—H1C	109.5	O1—C7—O2	124.6 (7)
N—C2—C3	121.4 (5)	O1—C7—C6	119.4 (7)
N—C2—C1	115.2 (5)	O2—C7—C6	115.9 (6)
C3—C2—C1	123.3 (6)	O2—C8—H8A	109.5
C4—C3—C2	120.4 (5)	O2—C8—H8B	109.5
C4—C3—Br	120.5 (5)	H8A—C8—H8B	109.5
C2—C3—Br	119.1 (5)	O2—C8—H8C	109.5
C5—C4—C3	117.9 (6)	H8A—C8—H8C	109.5
C5—C4—H4A	121.0	H8B—C8—H8C	109.5
			
C6—N—C2—C3	1.8 (10)	C2—N—C6—C7	177.4 (6)
C6—N—C2—C1	178.4 (6)	C4—C5—C6—N	0.4 (11)
N—C2—C3—C4	−2.7 (11)	C4—C5—C6—C7	−177.5 (7)
C1—C2—C3—C4	−179.0 (6)	C8—O2—C7—O1	−2.1 (11)
N—C2—C3—Br	179.6 (5)	C8—O2—C7—C6	175.0 (6)
C1—C2—C3—Br	3.3 (9)	N—C6—C7—O1	−167.1 (7)
C2—C3—C4—C5	2.3 (11)	C5—C6—C7—O1	11.0 (11)
Br—C3—C4—C5	180.0 (5)	N—C6—C7—O2	15.6 (9)
C3—C4—C5—C6	−1.2 (11)	C5—C6—C7—O2	−166.3 (7)
C2—N—C6—C5	−0.6 (10)		

Symmetry codes: (i) −*x*+1, *y*+1/2, −*z*+1/2.
